# Impact of *Plasmodium vivax* malaria on executive and cognitive functions in elderlies in the Brazilian Amazon

**DOI:** 10.1038/s41598-022-14175-0

**Published:** 2022-06-20

**Authors:** Rockson C. Pessoa, Gabriela F. Oliveira-Pessoa, Brenda K. A. Souza, Vanderson S. Sampaio, André Luiz C. B. Pinto, Larissa L. Barboza, Gabriel S. Mouta, Emanuelle Lira Silva, Gisely C. Melo, Wuelton M. Monteiro, José H. Silva-Filho, Marcus V. G. Lacerda, Djane Clarys Baía-da-Silva

**Affiliations:** 1 Programa de Pós-Graduação em Medicina Tropical, Universidade do Estado do Amazonas, Manaus, Brazil; 2 Instituto de Pesquisa Clínica Carlos Borborema, Fundação de Medicina Tropical Dr Heitor Vieira Dourado, Av Pedro Teixeira, 25, Manaus, Amazonas 69040-000 Brazil; 3 Instituto Leônidas & Maria Deane, Fundação Oswaldo Cruz, Manaus, Brazil; 4 Fundação de Vigilância em Saúde do Amazonas, Manaus, Brazil; 5 Universidade Federal do Amazonas, Manaus, Brazil

**Keywords:** Diseases, Infectious diseases, Malaria

## Abstract

The exact path leading to cognitive impairment that goes beyond malaria is unclear, but it appears to be the result of interactive factors. Time of exposure to disease and recurrences are potentially major determinant variables. Cognitive impairment is described mainly in children, rarely in adults. The disease in high endemic areas usually does not affect elderlies, because of acquired immunity over time. However, this population is relatively more frequently sick in lower endemic areas, such as in the Amazon. This study assessed the effect of *Plasmodium vivax* malaria on the executive and cognitive functions of elderlies, in the Brazilian Amazon. A cohort study was conducted to evaluate executive and cognitive functions one week (T0), two months (T2) and eight months (T8) after the malaria episode. Mini-Mental State Examination (MMSE), Beck Depression Inventory II (BDI-II), Clock Drawing Test (CDT), Wechsler adult intelligence scale (WAIS-III), and Wisconsin Card Sorting Test (WCST) were used to assess executive and cognitive functions. One hundred-forty elderlies were enrolled (70 with *P. vivax* malaria and 70 without malaria). *P. vivax* malaria was associated with impairment of the executive and cognitive functions in elderlies for up to 8 months after acute *P. vivax* malaria. Prior history of malaria, recurrences and higher parasitemia were independently associated with various surrogates of executive and cognitive impairment. With the increase in life expectancy, elderlies living in malaria endemic areas will deserve more attention from health authorities, to guarantee improvement of their quality of life in the tropics.

## Introduction

Approximately a third of the global population is at risk for *Plasmodium vivax* infection^1^. Although predominant globally, for a long time it was believed that *P. vivax* malaria was always associated with benign disease^2^. However, in recent decades, severe cases and deaths from *P. vivax* malaria have been reported^3^. The main complications of severe *P. vivax* malaria include pulmonary edema, severe acute respiratory syndrome, acute renal failure, severe anemia, hemorrhage, acidosis, and disseminated intravascular coagulation^4,5^. Cerebral malaria (CM), a potentially fatal condition associated with *P. falciparum* infection, has also been reported in *P. vivax* mono-infection in children, but is rare^6^.

A few studies address the association between malaria and cognition, particularly after the acute phase of the disease, during convalescence. Those few studies usually focus on cognition following the development of CM triggered by *P. falciparum* (CMF), which could be a potential cause of short- and long-term neurological and cognitive deficit in Subsaharan African children^7^. Few studies documented the effects of CMF on adults’ cognitive performance and none of these studies tested all the five facets of cognition (Working memory, planning, cognitive flexibility, inhibitory control and concept formation) making it difficult to identify patterns of cognitive impairment.

The exact path leading to cognitive impairment that goes beyond CM is unclear, but it appears to be the result of many potentially interactions. There are many risk factors, such as anemia, multiple infections, hippocampal dysfunction, damage to sub-cortical white matte, neurotoxins released from infected red blood cells, which may damage cortical areas of the brain, and cytokine storm^8,9^. Patterns of cognitive impairment may differ between children and adults, with age of exposure to disease being an important variable, as well as repetitive infections^10^.

Thus, although few studies evaluate the cognitive impairment of children after *P. vivax*^11–14^ or *P. falciparum* malaria^14–16^, in non-severe malaria, the impact on adults is not well addressed, especially in vivax malaria. Four studies investigated the impact of infection due to non-severe vivax malaria on cognition. A study conducted in Sri Lanka and determined the short-term impact of malaria on the cognitive performance of 571 schoolchildren (ages 1–8 years)^14^. Vitor-Silva et al.^11^, in a cross-sectional study carried out in the city of Careiro, Amazonas, with 198 students (aged 6–14 years) identified impairment in school performance. Brasil et al.^12^, in a study on the Marajó Island, Pará, with 17 schoolchildren (aged 2–10 years) demonstrated that children with a history of vivax malaria presented significant impairments in the cognitive, affective, instrumental domains (problem solving in activities of daily living) and in verbal comprehension (reasoning and abstraction centered on verbal comprehension and expression). Tapajós et al.^13^, in a study with 219 schoolchildren (aged 2–7 years) in the community of Brasileirinho, Manaus, Amazonas, revealed that vivax malaria is a risk factor for low cognitive development.

To our knowledge, there is no report on the assessment of cognition in elderlies after malarial infection, especially vivax malaria. This study assessed the effect of *P. vivax* malaria on executive and cognitive functions in such age group, in the Brazilian Amazon, where about 89,3% of malaria cases are caused by *P. vivax* in 2019^17^. Thus, due to the lack of knowledge on the subject and local epidemiological issues, it was decided to study exclusively *P. vivax* infection.

## Methods

### Ethical aspects

This study was approved by the Human Research Ethics Committee of *Fundação de Medicina Tropical Dr Heitor Vieira Dourado* (FMT-HVD) (CAAE: 71396317.7.0000.0005). All participants were informed about the objectives and risks of participation and signed informed consent terms. This study was conducted in accordance with the principles of the Declaration of Helsinki and the guidelines of Good Clinical Practice of the International Harmonization Conference.

### Study type

This was a cohort study, with a selection of malarial exposed (with malaria-cases) and non-exposed (without malaria-control), in which executive and cognitive functions were evaluated during an 8-month follow-up. Elderlies’ evaluations were performed during the first week after malaria infection (T0), two months (T2) and eight months (T8) later.

### Study location and population

The study was carried out from February 2018 to November 2020 at FMT-HVD, a reference institution for infectious diseases in Manaus, Amazonas State. After malaria diagnosis by thick blood smear, patients aged ≥ 60 years, both genders, were invited to participate. In case of interest in participating in the study, they received a domiciliary visit during the first week after the diagnosis. Vivax malaria patients were treated according to the Brazilian malaria treatment protocol^18^. The non-exposed group consisted of elderlies in the same age group, without malaria in the last six months of the interview, living in the same locality as the cases. Participants with any history of neurological or psychiatric impairment were not included in the study.

### Malaria and other diseases diagnosis

Thick blood smears were performed for the malaria diagnosis in all symptomatic patients^19^. All participants (exposed and unexposed) were instructed to seek the care unit if they manifested symptoms. In addition, all participants were followed in online official Malaria Epidemiological Surveillance Information System (Sivep-Malaria). At the time of assessment of executive and cognitive functions, in T2 and T8, peripheral venous blood was collected for assessment of asymptomatic infection by molecular techniques For DNA extraction, the miniblod Qiagen Kit was used, according to the manufacturer's instructions. Qmal Taqman PCR was used to detect *Plasmodium* spp. in DNA samples, as previously described^20^. Malaria recurrences over the study period have been confirmed in Sivep-Malária. Patients diagnosed with malaria were treated according to the Brazilian Ministry of Health's treatment guidelines.

Hepatitis C, HIV and syphilis were also investigated by serological rapid tests (Abbot) performed according to the manufacturer's recommended instructions, as potential concomitant causes of cognitive impairment at T0. At T2 and T8, blood samples were also collected to assess blood glucose and hemoglobin measurements, as uncontrolled diabetes and anemia are also potential confounders.

### Cognitive assessment

Participants answered to the socio-demographic cognition assessment form (Mini-Mental State Examination/MMSE and Clock Drawing Test/CDT) at T0. At T2 and T8, MMSE, CDT, Beck depression inventory II (BDI-II instruments), subtests (digits direct and indirect order, arithmetic and sequence of numbers and letters) of the Wechsler adult intelligence scale (WAIS-III), and Wisconsin Card Sorting Test (WCST) were applied. Global cognitive and executive functions were assessed by tests validated for the Brazilian population. The Addenbrooke’s cognitive examination III (ACE-III), a standard cognitive assessment, was undergoing validation during the study period^21^. Instruments used and their respective domains evaluated are summarized in Supplementary Table 1.

MMSE is an instrument that assesses mental state, more specifically the symptoms of dementia, through questions that assess orientation in time and place, registration and evocation of words, attention and ability to simple calculations, language, and visual constructive capacity^22^. BDI-II is a widely used self-report inventory measuring the severity of depression through 21-items^23^. CDT measures cognitive assessment and is used in the investigation of the impairment of some cognitive skills such as visual-constructive functions, visuo-spatial functions, symbolic and graphomotor representation, auditory language, semantic memory, and executive functions^24^.

The WAIS-III is an advanced cognitive assessment instrument used to assess intellectual ability in adults and is composed of several subtests that measure different aspects of intelligence^25^. As a measure of executive functions (EFs), the Working Memory Index (WMI) was used in these studies and was obtained through the weighted score of the subtests, digits in direct order and digits on the back, arithmetic and sequential numbers and letters. The correlation of their results with brain location has been the subject of many studies and has contributed significantly to the understanding of the action of the prefrontal cortex, specifically as dorsolateral and ventromedial subregions^26^.

WCST is an internationally recognized instrument for assessing EFs and is frequently adopted in neuropsychological assessments^27^. It evaluates strategic planning, organized search, the use of feedback from the environment to change cognitive strategies, the direction of behavior to achieve goals and the modulation of impulsive responses. The WCST assessment, therefore, can be used to assess and identify cognitive impairments and neurological conditions related to the frontal region of the brain that has deficits^27^. In this study, the WCST scores: "number of trials administered", "total number of errors"," trials to complete the first category", and "learning to learn", were not considered. The first three are redundant and the last one has the power to bias studies^28^.

### Sample size calculation

The sample size calculation assumed a 20% prevalence of cognitive impairment due to infection caused by *P. falciparum* in adults^29^. For the general population, the prevalence was assumed to be 6.1%^30^. Thus, considering a test of differences in proportions between two groups of the same size, 90% of power and 5% alpha error, 244 participants were required (122 per group). Adding 15% of losses, the final sample size was ~ 280 participants. Sample calculation was performed using software and R, version 4.0.5 (R project for Statistical Computing, http://www.R-project.org)^31^. Interim analysis was scheduled after the inclusion and follow-up of 140 patients.

### Statistical analysis

Normal distribution was assessed for variables with the Shapiro–Wilk test. Differences between baseline characteristics were evaluated by ANOVA, for continuous variables with normal distribution, Wilcoxon rank-sum test for continuous variables not normally distributed, and Pearson's Chi Squared test was applied for categorical variables. Multivariate analysis included age, sex, and education variables, as well as depression (expressed by BDI-II indicators).

The main and subgroup analyzes were performed with Poisson regression. The main analyzes compared executive and cognitive fucntions between subjects with malaria and without malaria. Additional subgroup analysis was performed for malaria patients considering three variables: (1) malaria episodes before enrollment; (2) recurrences between T0 and T8; and parasitemia higher than the median number of parasites/mm^3^. Previous univariate analysis was performed for multivariable variable selection. Variables with a p-value lower than 0.2 were included. All analyses were conducted in Stata 13.0 (Statacorp, 2013).

## Results

**Figure 1:**
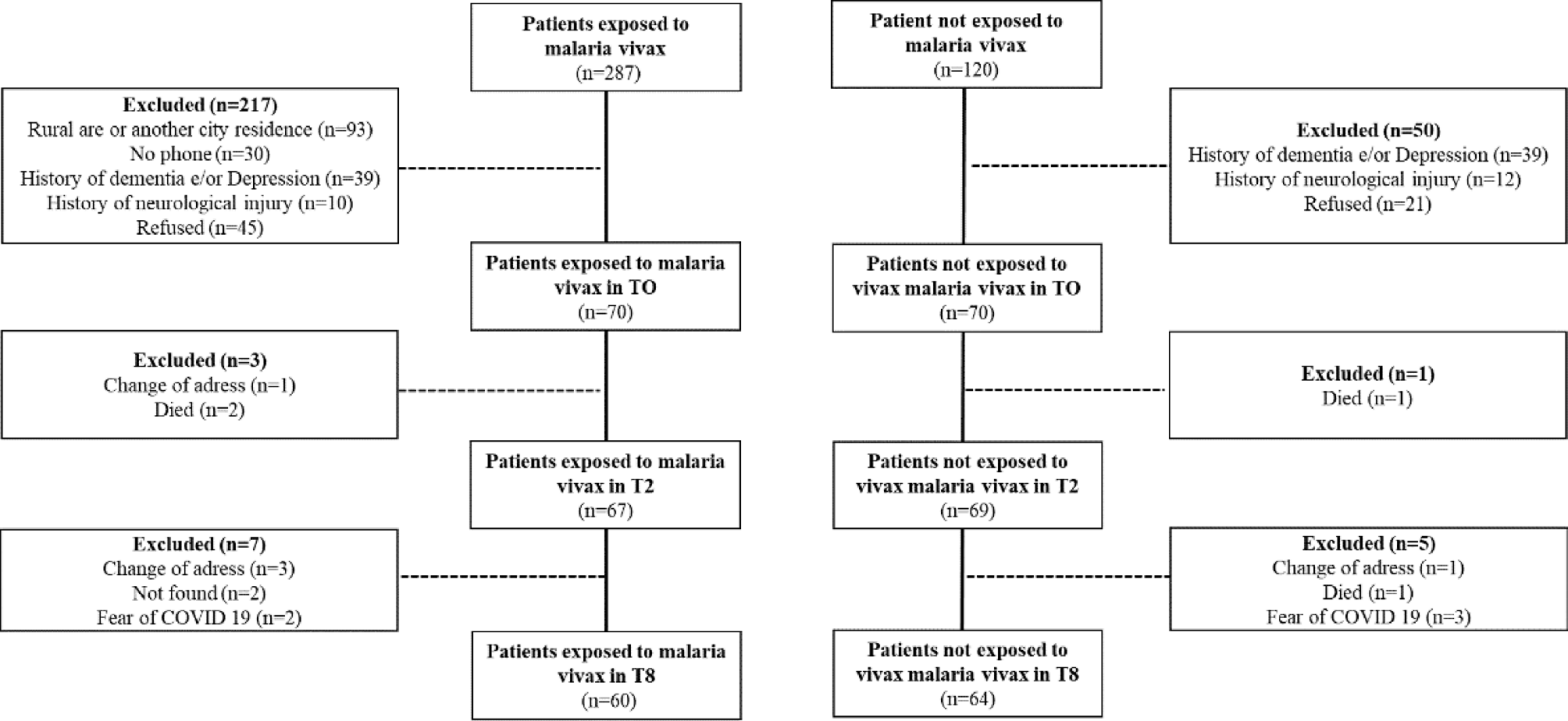
Flowchart of the study.

Out of 470 subjects, 140 were considered eligible (70 exposed and 70 non exposed) (Fig. 1). A total of 110 patients performed tests for HIV, syphilis and CHV (none was positive). Median age was 66 years (63.0–71.5). Women represented 51% from the total population. Exposed and no exposed differed in terms of age, sex, and education (Table 1).

**Table 1:** Demographic and clinical characteristics in the baseline.

Characteristics	Total N = 140	Non exposed to malaria n = 70	Exposed to malaria n = 70	*P*
Age, years, median (IQR)	66.0 (63.0–71.5)	69.0 (65.0–73.0)	65.0 (63.0–69.0)	**0.002**
**Gender, %**				**< 0.01**
Female	72/140 (51.4%)	46/70 (65.7%)	26/70 (37.1%)	
Male	68/140 (48.6%)	24/70 (34.3%)	44/70 (62.9%)	
**Schooling, %**				**0.013**
Illiterate	10/140 (7.1%)	2/70 (2.9%)	8/70 (11.4%)	
Primary	75/140 (53.6%)	32/70 (45.7%)	43/70 (61.4%)	
High school	32/140 (22.9%)	22/70 (31.4%)	10/70 (14.3%)	
University	23/140 (16.4%)	14/70 (20.0%)	9/70 (12.9%)	
Use of psychotropic, %	7/140 (5.0%)	3/70 (4.3%)	4/70 (5.7%)	0.70
**Marital status, %**				0.50
Married	87/140 (62.1%)	44/70 (62.9%)	43/70 (61.4%)	
Divorced	10/140 (7.1%)	7/70 (10.0%)	3/70 (4.3%)	
Widowed	22/140 (15.7%)	9/70 (12.9%)	13/70 (18.6%)	
Single	21/140 (15.0%)	10/70 (14.3%)	11/70 (15.7%)	
**Income, minimal wage, %**				0.20
1–2 MW	98/140 (70.0%)	45/70 (64.3%)	53/70 (75.7%)	
3–4 MW	28/140 (20.0%)	14/70 (20.0%)	14/70 (20.0%)	
5–6 MW	6/140 (4.3%)	5/70 (7.1%)	1/70 (1.4%)	
7–8 MW	6/140 (4.3%)	5/70 (7.1%)	1/70 (1.4%)	
Above 10 MW	2/140 (1.4%)	1/70 (1.4%)	1/70 (1.4%)	
**Occupation, %**				0.14
Retired	79/140 (56.4%)	43/70 (61.4%)	36/70 (51.4%)	
Pensioner	11/140 (7.9%)	7/70 (10.0%)	4/70 (5.7%)	
Self-employed	7/140 (5.0%)	1/70 (1.4%)	6/70 (8.6%)	
Housewife	10/140 (7.1%)	6/70 (8.6%)	4/70 (5.7%)	
Never worked	33/140 (23.6%)	13/70 (18.6%)	20/70 (28.6%)	
**Residence type, %**				0.14
Own	126/139 (90.6%)	62/69 (89.9%)	64/70 (91.4%)	
Rented	6/139 (4.3%)	5/69 (7.2%)	1/70 (1.4%)	
Family residence	7/139 (5.0%)	2/69 (2.9%)	5/70 (7.1%)	
Leisure, activities, median (IQR)	3.0 (2.0–5.0)	3.5 (2.0–5.0)	3.0 (2.0–5.0)	0.051
**Beck Depression Inventory (BDI), points, median (IQR)**				
At T2	3.0 (1.5–6.0)	3.0 (1.0–5.0)	4.0 (2.0–7.0)	0.23
At T8	4.5 (2.0–7.0)	5.0 (3.0–8.0)	4.0 (2.0–7.0)	0.13
**Hemoglobin, g/dL**				
At T2, median (IQR)	10.9 (10.1–12.1)	10.6 (10.0–12.0)	11.2 (10.2–12.1)	0.22
At T8, mean (SD)	11.2 (1.9)	11.0 (2.0)	11.3 (1.7)	0.39

Significant values are in bold.

According to official information system, no previous malaria infections were reported in the control group, while 57.1% of cases had previous episodes in the period. In the case group, 75.7% did not present recurrences of malaria until assessment in T8, and 69.1% had higher than 500 parasites/mm^3^ parasitaemia at T0 infection (Supplementary Table 2). At T2 and T8, no parasites were identified in the blood smear.

**Figure 2:**
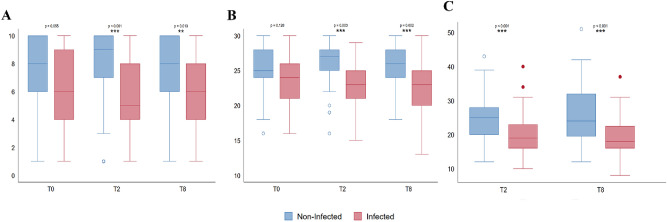
Cognitive (**A**,**B**) and executive performance (**C**) assessed by Clock Drawing Test (**A**), Mini-Mental State Examination (**B**) and WAIS-III (**C**).

**Figure 3:**
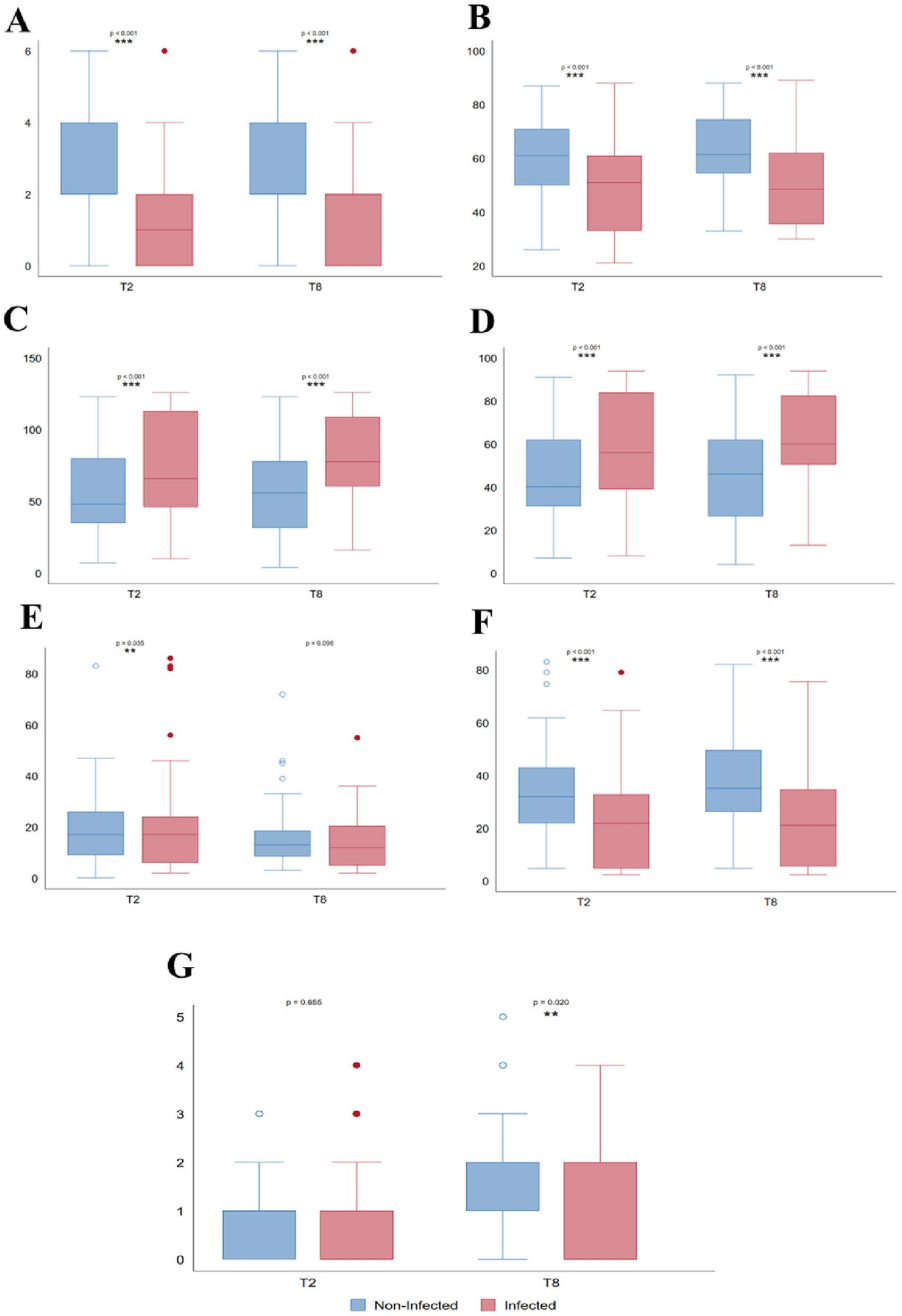
Executive performance assessed by WCST indicators. (**A**) Number of categories completed, (**B**) number total corrects (**C**) perseverative responses, (**D**) perseverative errors, (**E**) nonperseverative errors, (**F**) percent conceptual level responses and (**G**) failure to maintain set.

In univariable analysis, exposed group’s performance was worse than non-exposed group at T0 assessment (CDT: p < 0.01; MMSE: p = 0.03). However, in the multivariate analysis, no differences were found (CDT: p = 0.055; MMSE: p = 0.128), even though the marginal significance indicated a trend in worst performance in the exposed group. Otherwise, the malaria group had worse performance in cognitive (CDT: T2 p < 0.01; T8 p < 0.05; MMSE: T2 p < 0.05; T8 p < 0.05) and executive functions (WAIS: T2 p < 0.01; T8 p < 0.01; WCST categories: T2 p < 0.05; T8 p < 0.05), both at T2 and T8, independently of age, sex, education and depression (Supplementary Table 3, Figs. 2 and 3).

Higher parasitemia at T0, higher number of previous malaria infections and recurrent malaria after T0 had a negative impact on cognition and executive functions at T2 (WCST categories: p < 0.05) and T8 (WCST categories: p < 0.05) (Supplementary Tables 4, 5 and 6).

## Discussion

This cohort study is one of the first attempts to examine the association of *P. vivax* malaria between executive and cognitive functions in elderlies. The findings suggest that elderlies with *P. vivax* malaria experienced deficits in their executive and cognitive functions from 2 months to at least 8 months post-infection. Higher parasitemia, prior and recurrent malaria negatively impacted executive functions in the elderlies. Thus, given the high number of *P. vivax* infections and an ageing population worldwide, the magnitude of this problem might not be negligible. The recognition of cognitive deficits and variability resulting from ageing, infectious diseases, or metabolic processes, requires early identification since there is a risk of evolution to dementia and a negative impact on the quality of life in the elderlies and their families^32–36^. Poorer cognitive function is associated with an increased risk of depression, social withdrawal, and dependence, and may contribute to decreased quality of life^33,36^.

The impact of malarial infections on cognition has been assessed in severe and non-severe falciparum and vivax malaria, and the studies are almost exclusively performed in children^11–16,37^. These studies show that malaria affects language development, attention span in children and adolescents and negatively impacts their cognitive development and school performance. Cognitive and executive losses associated with infectious diseases are poorly studied amongst elderlies. This lack of studies may be related to the difficulty in establishing whether the losses are associated with senility or to the infectious process per se. However, some infectious agents have been thought as potential factors contributing to dementia^38,39^. The clinical pattern of malaria, especially severe malaria, differs between children and adults. However, it is uncertain whether these differences reflect the age of affected individuals or other differences between populations in the characteristics of host, parasite, pattern of exposure or provision of health services^40^. These factors may interfere in distinguishing the cognitive effects associated with malaria between children and the elderly, however it is important to note that the impact of cognitive impairment on the child's life may be more significant, as it reflects on delays and important losses in school development, especially, that can be reflected throughout their adult life^7,11–16,41^.

Executive and cognitive assessment is a valuable clinical tool and usually involves the use of two types of psychological instruments or tests (screening tests and diagnostic or confirmatory tests). In this study, we used tools with different sensitivities, which resulted in different results regarding the determination of cognitive and executive functions. However, it is important to emphasize that screening tests MMSE and CDT have high specificity and are used to signal mild cognitive impairment and a maximum number of suspected psychological processes and are suitable to safely exclude false positives. The other tests used WCST and (WAIS/WMI) are high sensitivity instruments and used to confirm any psychological condition and confirm true positives, and are used to signal mild cognitive impairment and a maximum number of suspected psychological processes^42^.

Infections by herpes simplex virus type 1 (HSV-1)^43^ and cytomegalovirus (CMV)^39^, HIV^44^, and bacteria, such as *Chlamydia pneumoniae*^45^ and *Helicobacter pylori*^46^ and *Lyme neuroborreliosis*^47^ have been associated with cognitive impairment. In older adults with pneumonia and urinary tract infection, for example, the risk of cognitive loss is about 1.4 times higher and previous episodes are positively associated with cognitive loss^48^. Inflammatory reactions have been highly related to cognitive decline and risk of dementia^49^. Inflammatory cytokines produced by the nervous system act on neural substrates to produce behavioral symptoms that can be related to cognitive changes^50^. Thus, like the diseases, vivax malaria is capable of significantly altering cognitive and executing functions for up to 8 months, which, in turn, may affect the quality of life of the elderly.

In children, parasitemia, the number of previous and recurrent malaria has a negative impact on cognition and executive functions, with a cumulative negative effect on school performance being observed^7,11–16,41^. In the elderlies, we observed similar effects up to 8 months after the malaria episode. Parasitemia at T0, number of previous and recurrent malaria had a negative impact on cognition and executive functions.

The cognitive mechanisms underlying malaria remain poorly understood and experimental models of severe malaria, particularly the murine model using the *Plasmodium berghei* ANKA (PBA) strain, are a valuable tool for understanding the cognitive and neurological outcomes associated with this condition^51,52^. Studies with an animal model are focused on seeking to understand mainly the cognitive and neurological mechanisms, based on the implementation of CM. Neuroinflammation after PBA infection of mice influences neurotrophin expression, which impairs hippocampal neurogenesis and increases hippocampal cell death. This is associated with impaired memory, but specifically short-term memory after the CM course^51^. Inflammation of the central nervous system, because of CM, determines the release of inflammatory cytokines, which affect neurogenesis and reverberate in cognitive defects^53^. The study organized by Azevedo-Quintanilha et al. revealed that αDβ2 integrin deletion alters the natural course of experimental severe malaria, demonstrating previously unrecognized activities of a key leukocyte integrin in the immunoinflammatory responses that mediate brain involvement^54^. In non-severe malaria, the cognitive impairment mechanisms are not known, but they may be associated with cytokine storms during acute infection^55^, the production of neurotoxins by infected red blood cells^56^, impaired coagulation that can impact the correct oxygen supplementation to the tissues of the central nervous system^57^, anemia^58^, multiple infections^59^.

*Plasmodium vivax* can be associated with neurological and vascular impairment^60^. The predilection for reticulocytes, marked proinflammatory responses, and the reversible microvascular dysfunction typically associated with *P. vivax* infection may explain the pathogeny of CM in malaria vivax^61–63^. Vascular congestion, hypoperfusion, and localized hypoxia in CM occurs mainly in the occipital and parietal lobes, this in turn has important reflexes in visuospatial deficit^64–67^. In the present work, visuospatial deficits were evaluated with the MMSE and CDT tools and the exposed group's performance was worse than non-exposed group. Executive functions and visuospatial skills are two interrelated cognitive processes and these deficits, which are associated with a decline in the performance of daily activities^68,69^.

It must be considered that chloroquine (CQ) used for the treatment of vivax malaria may have as an adverse event psychiatric and nervous system disorders, which may reflect on changes in cognitive and executive functions during the treatment of vivax malaria^70,71^. Since only the patients exposed in this study had previous malaria, they are in an endemic area, where CQ has been the treatment of first choice for many years and low CQ resistance is defined, we consider that CQ may potentially play an important role in altering neurocognitive functions. CQ is reported as an inhibitor of autophagy in a variety of diseases, including Alzheimer's disease and cerebral ischemia. After inducing brain trauma, treatment with CQ significantly suppressed neuronal autophagy and reduced levels of expression of inflammatory cytokines, interleukin-1β (IL-1β) and tumour necrosis factor-α (TNF-α), in the hippocampus of the mouse^72^ and this can reduce cognitive impairment. In contrast, patients treated with CQ (cases) showed worsening cognition and executing functions. However, the half-life of the drug is highly unlikely to explain the long-term cognitive impairment seen in our patients at T8.

In this study, we did not assess other potential co-infections associated with malaria or underlying diseases that could potentially interfere with cognition. A systematic approach to addressing these confounders would be important, however, the clinical and pre-clinical follow-up of these patients to define potential confounders was hampered in this study population, especially due to home care and to the fact that they present at the hospital unit exclusively for the malaria diagnosis. The use of secondary information from SIVEP is also an important limiting factor. Although the groups exposed and not exposed to malaria were selected from the same transmission area, other variables were not properly paired. The adjustment of variables not properly paired were included in multivariate analyzes and had no impact on cognition alone. Attention and language were evaluated by instruments of low sensitivity.

## Concluding remarks

Our results suggest that malaria promotes cognitive, executive, and functional decline in the elderly population for up to 8 months after *P. vivax* infection, and that factors such as number of previous malaria cases, recurrence, and parasitemia are important predictors of executive impairment in the second and eighth months after infection. In the context of a rapidly ageing population and the high rates of transmission of vivax malaria in subtropical and tropical areas, where vivax malaria is endemic, malaria can contribute with a substantial negative impact on cognition and executing functions that reflect on the life quality. This, in turn, adds to the deleterious events of the normal ageing process (the lack of health guarantee and the risks of developing diseases common to this stage of life, such as dementia). The revelations obtained in this study should serve as a notice to the policy makers and professionals involved in the healthcare and improvement of the elderlies’ quality of life, considering that this population is vulnerable to cognitive damage.

### Supplementary Information

Supplementary Tables.

## Abbreviations

MMSE: Mini Mental State Examination
BDI-II: Beck Depression Inventory-II
CDT: Clock Drawing Test
WAIS-III: Wechsler Adult Intelligence Scale—Third Edition
WCST: Wisconsin Card Sorting Test
CM: Cerebral malaria
CMf: Cerebral malaria caused by *Plasmodium falciparum*
PCR: Polymerase chain reaction
DNA: Deoxyribonucleic acid; WISC
EFs: Executive functions
