# Author Correction: Impact of *Plasmodium vivax* malaria on executive and cognitive functions in elderlies in the Brazilian Amazon

**DOI:** 10.1038/s41598-023-48865-0

**Published:** 2024-01-11

**Authors:** Rockson C. Pessoa, Gabriela F. Oliveira-Pessoa, Brenda K. A. Souza, Vanderson S. Sampaio, André Luiz C. B. Pinto, Larissa L. Barboza, Gabriel S. Mouta, Emanuelle Lira Silva, Gisely C. Melo, Wuelton M. Monteiro, José H. Silva-Filho, Marcus V. G. Lacerda, Djane Clarys Baía-da-Silva

**Affiliations:** 1 Programa de Pós-Graduação em Medicina Tropical, Universidade do Estado do Amazonas, Manaus, Brazil; 2 Instituto de Pesquisa Clínica Carlos Borborema, Fundação de Medicina Tropical Dr Heitor Vieira Dourado, Av Pedro Teixeira, 25, Manaus, Amazonas 69040-000 Brazil; 3 Instituto Leônidas & Maria Deane, Fundação Oswaldo Cruz, Manaus, Brazil; 4 Fundação de Vigilância em Saúde do Amazonas, Manaus, Brazil; 5 Universidade Federal do Amazonas, Manaus, Brazil

Correction to: *Scientific Reports* 10.1038/s41598-022-14175-0, published online 20 June 2022

Due to issues with automatic referencing software, this Article contained errors in the Reference list.

In the Introduction, the following sentences contained errors.

“Approximately a third of the global population is at risk for Plasmodium vivax infection^1^.”

Reference 1 was incorrectly given as:

Rangel, G. W. *et al.* *Plasmodium vivax* transcriptional profiling of low input cryopreserved isolates through the intraerythrocytic development cycle. *PLoS Negl. Trop. Dis.* **14**(3), 1–18 (2020).

The correct reference is listed below:

Battle, K. E. & Baird, J. K. The global burden of *Plasmodium vivax* malaria is obscure and insidious. *PLoS Med*. **18**(10): e1003799 10.1371/journal.pmed.1003799 (2021).

“However, in recent decades, severe cases and deaths from *P. vivax* malaria have been reported^3^.”

Reference 3 was incorrectly given as:

Fernandez-Becerra, C., Bernabeu, M., Castellanos, A., Correa, B. R., Obadia, T., Ramirez, M. *et al*. *Plasmodium vivax* spleen-dependent genes encode antigens associated with cytoadhesion and clinical protection. *Proc. Natl. Acad. Sci. U. S. A.* **117**(23), 13056–13065 10.1073/pnas.1920596117 (2020).

The correct reference is listed below:

Naing, C., Whittaker, M. A., Nyunt Wai, V. & Mak, J. W. Is *Plasmodium vivax* malaria a severe malaria?: A systematic review and meta-analysis. *PLoS Negl. Trop. Dis*.** 8**(8), e3071 10.1371/journal.pntd.0003071 (2014).

“There are many risk factors, such as anemia, multiple infections, hippocampal dysfunction, damage to sub-cortical white matte, neurotoxins released from infected red blood cells, which may damage cortical areas of the brain, and cytokine storm^8^.”

Reference 8 was incorrectly given as:

Nimgaonkar, V. L. *et al.* Temporal cognitive decline associated with exposure to infectious agents in a population-based, aging cohort. *Alzheimer Dis. Assoc. Disord.* **30**(3), 216–222 (2016).

The correct references are listed below:

Milner Jr, D. A., Whitten, R. O., Kamiza, S., Carr, R., Liomba, G., Dzamalala, C., Seydel, K. B., Molyneux, M. E. & Taylor, T. E. The systemic pathology of cerebral malaria in African children. *Front. Cell Infect. Microbiol*. **4**, 104 10.3389/fcimb.2014.00104 (2014).

Idro, R., Marsh, K., John, C. *et al*. Cerebral malaria: Mechanisms of brain injury and strategies for improved neurocognitive outcome. *Pediatr. Res*. **68**, 267–274 10.1203/PDR.0b013e3181eee738 (2010).

“Thus, although few studies evaluate the cognitive impairment of children after *P. vivax*^13,14^ or *P. falciparum* malaria^15,16^ (…)”

References 13 and 14 were incorrectly given as:

Bangirana, P. *et al.* Malaria with neurological involvement in Ugandan children: Effect on cognitive ability, academic achievement and behaviour. *Malar. J.* **10**(1), 334 10.1186/1475-2875-10-334 (2011).

Ministério da Saúde. Boletim Epidemiológico Malária. Secretaria de Vigilância em Saúde. Brasília. *Epidemiol. Rep.* 1–118. https://www.gov.br/saude/pt-br/media/pdf/2020/dezembro/03/boletim_especial_malaria_1dez20_final.pdf (2020). Accessed 20 Aug 2021.

The correct references are listed below:

Vitor-Silva, S., Reyes-Lecca, R.C., Pinheiro, T.R. & Lacerda, M.V. Malaria is associated with poor school performance in an endemic area of the Brazilian Amazon. *Malar J*. **8**, 230 10.1186/1475-2875-8-230 (2009).

Brasil, L. M. B. F., Vieira, J. L. F., Araújo, E. C., Piani, P. P. F., Dias, R. M., Ventura, A. M. R. S., Cabral, B. C., Santa Brígida, R. C. R. & de Andrade, M. A. Cognitive performance of children living in endemic areas for *Plasmodium vivax*. *Malar. J*. **16**(1), 370 10.1186/s12936-017-2026-2 (2017).

Additionally, the Authors omitted the below References:

Tapajós, R., Castro, D., Melo, G., Balogun, S., James, M., Pessoa, R., Almeida, A., Costa, M., Pinto, R., Albuquerque, B., Monteiro, W., Braga, J., Lacerda, M. & Mourão, M.P. Malaria impact on cognitive function of children in a peri-urban community in the Brazilian Amazon. *Malar J*. **18**(1), 173 10.1186/s12936-019-2802-2 (2019).

Fernando, S.D., Gunawardena, D.M., Bandara, M.R., De Silva, D, Carter, R., Mendis, K.N. & Wickremasinghe, A.R. The impact of repeated malaria attacks on the school performance of children. *Am. J. Trop. Med. Hyg*. **69**(6), 582–588 (2003).

Furthermore, References 15 and 16 were incorrectly given as:

da Brasil, M. S. *Guia de Tratamento da Malária no Brasil*. http://www.fmt.am.gov.br/gabinete/uploads/guia-tratamento-malaria-preliminar-2019.pdf (2019). Accessed 20 Aug 2021.

Almeida, A. C. G. *et al.* High proportions of asymptomatic and submicroscopic *Plasmodium vivax* infections in a peri-urban area of low transmission in the Brazilian Amazon. *Parasit. Vectors 11*(1), 194 10.1186/s13071-018-2787-7 (2018).

The correct references are listed below:

Fernando, S. D., Gunawardena, D. M., Bandara, M. R., De Silva, D., Carter, R., Mendis, K. N. & Wickremasinghe, A. R. The impact of repeated malaria attacks on the school performance of children. *Am. J. Trop. Med. Hyg*. **69**(6), 582–588 (2003).

Halliday, K. E., Karanja, P., Turner, E. L., Okello, G., Njagi, K., Dubeck, M. M., Allen, E., Jukes, M. C. & Brooker, S. J. *Plasmodium falciparum*, anaemia and cognitive and educational performance among school children in an area of moderate malaria transmission: Baseline results of a cluster randomized trial on the coast of Kenya. *Trop. Med. Int. Health* **17**(5), 532–549 10.1111/j.1365-3156.2012.02971 (2012).

In addition, the Authors omitted the below References:

Carter, J. A., Mung'ala-Odera, V., Neville, B. G., Murira, G., Mturi, N., Musumba, C. & Newton, C. R. Persistent neurocognitive impairments associated with severe falciparum malaria in Kenyan children. *J. Neurol. Neurosurg. Psychiatry* **76**(4), 476–481 (2005).

SVS/MS. *Manual de Diagnostico Laboratorial da Malaria*. 112 (2009).

“A study conducted in Sri Lanka and determined the short-term impact of malaria on the cognitive performance of 571 schoolchildren (ages 1–8 years)^10^.”

Reference 10 was incorrectly given as:

Zuanetti, P., Dornelas, R. & Guedes-granzotti, R. B. Caracterização da memória de adultos. *Distúrbios Comun.* **29**(2), 218–226 (2017).

The correct reference is listed below:

Fernando, S. D., Gunawardena, D. M., Bandara, M. R., De Silva, D., Carter, R., Mendis, K. N. & Wickremasinghe, A. R. The impact of repeated malaria attacks on the school performance of children. *Am. J. Trop. Med. Hyg*. **69**(6), 582–588 (2003).

“Oliveira-Ferreira et al.^11^, in a cross-sectional study carried out in the city of Careiro, Amazonas, with 198 students (aged 6–14 years) identified impairment in school performance.”

Reference 11 was incorrectly given as:

Guo, Y. Cognitive analysis of long-term memory in interpreting. *Int. J. Lang. Linguist.* **4**(3), 103–113 (2016).

The correct reference is listed below:

Vitor-Silva, S., Reyes-Lecca, R. C., Pinheiro, T. R. & Lacerda, M. V. Malaria is associated with poor school performance in an endemic area of the Brazilian Amazon. *Malar J*. **8**, 230 10.1186/1475-2875-8-230 (2009).

“Tapajós et al.^13^, in a study with 219 schoolchildren (aged 2–7 years) in the community of Brasileirinho, Manaus, Amazonas, revealed that vivax malaria is a risk factor for low cognitive development.”

Reference 13 was incorrectly given as:

Bangirana, P. *et al.* Malaria with neurological involvement in Ugandan children: Effect on cognitive ability, academic achievement and behaviour. *Malar. J.* **10**(1), 334 10.1186/1475-2875-10-334 (2011).

The correct reference is listed below:

Tapajós, R., Castro, D., Melo, G., Balogun, S., James, M., Pessoa, R., Almeida, A., Costa, M., Pinto, R., Albuquerque, B., Monteiro, W., Braga, J., Lacerda, M. & Mourão, M. P. Malaria impact on cognitive function of children in a peri-urban community in the Brazilian Amazon. *Malar. J*. **18**(1), 173 10.1186/s12936-019-2802-2 (2019).

“Brasil et al.^12^, in a study on the Marajó Island, Pará, with 17 schoolchildren (aged 2–10 years) demonstrated that children with a history of vivax malaria presented significant impairments in the cognitive, affective, instrumental domains (problem solving in activities of daily living) and in verbal comprehension (reasoning and abstraction centered on verbal comprehension and expression).”

Reference 12 was incorrectly given as:

Zanella, L. W. & Valentini, N. C. Como funciona a Memória de Trabalho? Influências na aprendizagem de crianças com dificuldades de aprendizagem e crianças com desordem coordenativa desenvolvimental (AU) TT—How the working memory functioning? Influences in learning of children with typi. *Medicine *(*Ribeiräo Preto*)* 49*(2), 160–174 (2016).

The correct reference is listed below:

Brasil, L. M. B. F., Vieira, J. L. F., Araújo, E. C., Piani, P. P. F., Dias, R. M., Ventura, A. M. R. S., Cabral, B.C., Santa Brígida, R. C. R. & de Andrade, M. A. Cognitive performance of children living in endemic areas for *Plasmodium vivax*. *Malar. J*. **16**(1), 370. 10.1186/s12936-017-2026-2 (2017).

In the Methods section, under the subheading ‘Malaria and other diseases diagnosis’,

“Thick blood smears were performed for the malaria diagnosis in all symptomatic patients^1^.”

Reference 1 was incorrectly given as:

Rangel, G. W. *et al.* *Plasmodium vivax* transcriptional profiling of low input cryopreserved isolates through the intraerythrocytic development cycle. *PLoS Negl. Trop. Dis.* **14**(3), 1–18 (2020).

The correct reference is listed below:

SVS/MS. *Manual de Diagnostico Laboratorial da Malaria*. 112 (2009).

In the Discussion section,

“The impact of malarial infections on cognition has been assessed in severe and non-severe falciparum and vivax malaria, and the studies are almost exclusively performed in children^33^”

Reference 33 was incorrectly given as:

Cohee, L. M. *et al.* Preventive malaria treatment among school-aged children in sub-Saharan Africa: A systematic review and meta-analyses. *Lancet Glob. Health.* **8**(12), e1499–e1511 (2020).

The correct reference is listed below:

Vitor-Silva, S., Reyes-Lecca, R. C., Pinheiro, T. R. & Lacerda, M. V. Malaria is associated with poor school performance in an endemic area of the Brazilian Amazon. *Malar. J*. **8**, 230 10.1186/1475-2875-8-230 (2009).

Additionally, the Authors omitted the below References:

Tapajós, R., Castro, D., Melo, G., Balogun, S., James, M., Pessoa, R., Almeida, A., Costa, M., Pinto, R., Albuquerque, B., Monteiro, W., Braga, J., Lacerda, M. & Mourão, M. P. Malaria impact on cognitive function of children in a peri-urban community in the Brazilian Amazon. *Malar. J*. **18**(1), 173 10.1186/s12936-019-2802-2 (2019).

Brasil, L. M. B. F., Vieira, J. L. F., Araújo, E. C., Piani, P. P. F., Dias, R. M., Ventura, A. M. R. S., Cabral, B. C., Santa Brígida R. C. R. & de Andrade, M. A. Cognitive performance of children living in endemic areas for *Plasmodium vivax*. *Malar. J*. **16**(1), 370 10.1186/s12936-017-2026-2 (2017).

Fernando, S. D., Gunawardena, D. M., Bandara, M. R., De Silva, D., Carter, R., Mendis, K. N. & Wickremasinghe, A. R. The impact of repeated malaria attacks on the school performance of children. *Am. J. Trop. Med. Hyg*. **69**(6), 582–588 (2003).

Halliday, K. E., Karanja, P., Turner, E. L., Okello, G., Njagi, K., Dubeck, M. M., Allen, E., Jukes, M. C. & Brooker, S. J. *Plasmodium falciparum*, anaemia and cognitive and educational performance among school children in an area of moderate malaria transmission: Baseline results of a cluster randomized trial on the coast of Kenya. *Trop. Med. Int. Health* **17**(5), 532–549 10.1111/j.1365-3156.2012.02971 (2012).

Carter, J. A., Mung'ala-Odera, V., Neville, B. G., Murira, G., Mturi, N., Musumba, C. & Newton, C. R. Persistent neurocognitive impairments associated with severe falciparum malaria in Kenyan children. *J. Neurol. Neurosurg. Psychiatry* **76**(4), 476–481 (2005).

Furthermore, References 7 and 35 in the Discussion section were removed.

“These factors may interfere in distinguishing the cognitive effects associated with malaria between children and the elderly, however it is important to note that the impact of cognitive impairment on the child's life may be more significant, as it reflects on delays and important losses in school development, especially, that can be reflected throughout their adult life^10–13,19–22,39^.”

References 10 and 11 were incorrectly given as:

Zuanetti, P., Dornelas, R. & Guedes-granzotti, R. B. Caracterização da memória de adultos. *Distúrbios Comun.* **29**(2), 218–226 (2017).

Guo, Y. Cognitive analysis of long-term memory in interpreting. *Int. J. Lang. Linguist.* **4**(3), 103–113 (2016).

The correct references are listed below:

Vitor-Silva, S., Reyes-Lecca, R. C., Pinheiro, T. R. & Lacerda, M. V. Malaria is associated with poor school performance in an endemic area of the Brazilian Amazon. *Malar J*. **8**, 230 10.1186/1475-2875-8-230 (2009).

Tapajós, R., Castro, D., Melo, G., Balogun, S., James, M., Pessoa, R., Almeida, A., Costa, M., Pinto, R., Albuquerque, B., Monteiro, W., Braga, J., Lacerda, M. & Mourão, M. P. Malaria impact on cognitive function of children in a peri-urban community in the Brazilian Amazon. *Malar. J*. **18**(1), 173 10.1186/s12936-019-2802-2 (2019).

References 12 and 19 were incorrectly given as:

Zanella, L. W. & Valentini, N. C. Como funciona a Memória de Trabalho? Influências na aprendizagem de crianças com dificuldades de aprendizagem e crianças com desordem coordenativa desenvolvimental (AU) TT—How the working memory functioning? Influences in learning of children with typi. *Medicine *(*Ribeiräo Preto*)* 49*(2), 160–174 (2016).

Byrne, B. M., Stewart, S. M. & Lee, P. W. H. Validating the Beck Depression Inventory-II for Hong Kong community adolescents. *Int. J. Test.* **4**(3), 199–216 10.1207/s15327574ijt0403_1 (2004).

The correct references are listed below:

Brasil, L. M. B. F., Vieira, J. L. F., Araújo, E. C., Piani, P. P. F., Dias, R. M., Ventura, A. M. R. S., Cabral, B.C., Santa Brígida, R. C. R. & de Andrade, M. A. Cognitive performance of children living in endemic areas for *Plasmodium vivax*. *Malar. J*. **16**(1), 370 10.1186/s12936-017-2026-2 (2017).

Fernando, S. D., Gunawardena, D. M., Bandara, M. R., De Silva, D., Carter, R., Mendis, K. N. & Wickremasinghe, A. R. The impact of repeated malaria attacks on the school performance of children. *Am. J. Trop. Med. Hyg*. **69**(6), 582–588 (2003).

References 20 and 21 were incorrectly given as:

Meagher, D. *et al.* A systematic review and meta-analysis of the accuracy of the clock drawing test (CDT) in the identification of delirium in older hospitalised patients. *Aging Ment. Health *10.1080/13607863.2020.1727849 (2020).

Wechsler, D. *WAIS-III Wechsler Adult Intelligence Scale*. 3rd edn (ed Corporation, P.) (1997).

The correct references are listed below:

Halliday, K. E., Karanja, P., Turner, E. L., Okello, G., Njagi, K., Dubeck, M. M., Allen, E., Jukes, M. C. & Brooker, S. J. *Plasmodium falciparum*, anaemia and cognitive and educational performance among school children in an area of moderate malaria transmission: Baseline results of a cluster randomized trial on the coast of Kenya. *Trop. Med. Int. Health* **17**(5), 532–549 10.1111/j.1365-3156.2012.02971 (2012).

Carter, J. A., Mung'ala-Odera, V., Neville, B. G., Murira, G., Mturi, N., Musumba, C. & Newton, C. R. Persistent neurocognitive impairments associated with severe falciparum malaria in Kenyan children. *J. Neurol. Neurosurg. Psychiatry* **76**(4), 476–481 (2005).

References 22 and 39 were incorrectly given as:

Strauss, E. & Sherman, E. M. S. *A Compendium of Neuropsychological Tests: Administration, Norms, and Commentary*. 3rd edn (ed O. U. Press) (Oxônia, Reino Unido, 1998).

Venkataramani, A. S. Early life exposure to malaria and cognition in adulthood: Evidence from Mexico. *J. Health Econ.* **31**(5), 767–780 10.1016/j.jhealeco.2012.06.003 (2012).

The correct references are listed below:

Vorasan, N., Pan-Ngum, W., Jittamala, P., Maneeboonyang, W., Rukmanee, P. & Lawpoolsri, S. Long-term impact of childhood malaria infection on school performance among school children in a malaria endemic area along the Thai-Myanmar border. *Malar. J.* **14**(1), 401 10.1186/s12936-015-0917-7 (2015).

Ssenkusu, J. M., Hodges, J. S., Opoka, R. O., Idro, R., Shapiro, E., John, C. C. & Bangirana, P. Long-term behavioral problems in children with severe malaria. *Pediatrics* **138**(5), e20161965 10.1542/peds.2016-1965 (2016).

“Infections by herpes simplex virus type 1 (HSV-1)^8^ and cytomegalovirus (CMV)^37^, HIV^41^, and bacteria, such as *Chlamydia pneumoniae*^8*^ and *Helicobacter pylori*^8^ and *Lyme neuroborreliosis*^42^ have been associated with cognitive impairment.”

Reference 8 in “[…] such as *Chlamydia pneumoniae*^8*^ and *Helicobacter pylori*^8^ […]” was incorrectly given as:

Nimgaonkar, V. L. *et al.* Temporal cognitive decline associated with exposure to infectious agents in a population-based, aging cohort. *Alzheimer Dis. Assoc. Disord.* **30**(3), 216–222 (2016).

The correct references are listed below:

Gérard, H. C., Dreses-Werringloer, U., Wildt, K. S., Deka, S., Oszust, C., Balin B. J., Frey 2nd, W. H., Bordayo, E. Z., Whittum-Hudson, J. A. & Hudson, A. P. *Chlamydophila* (*Chlamydia*) *pneumoniae* in the Alzheimer's brain. *FEMS Immunol. Med. Microbiol*. **48**(3), 355–366 10.1111/j.1574-695X.2006.00154.x (2006).

Tsolaki, F., Kountouras, J., Topouzis, F. & Tsolaki, M. *Helicobacter pylori* infection, dementia and primary open-angle glaucoma: Are they connected? *BMC Ophthalmol*. **15**, 24 10.1186/s12886-015-0006-2 (2015).

“In children, parasitemia, the number of previous and recurrent malaria has a negative impact on cognition and executive functions, with a cumulative negative effect on school performance being observed^20,46^.”

References 20 and 46 were incorrectly given as:

Meagher, D. *et al.* A systematic review and meta-analysis of the accuracy of the clock drawing test (CDT) in the identification of delirium in older hospitalised patients. *Aging Ment. Health* 10.1080/13607863.2020.1727849 (2020).

Ralano, A. A. H. A. Genetic influences on frontal brain function: WCST performance in twins. *NeuroReport* **14**(15), 1975–1978 (2003).

The correct references are listed below:

Vitor-Silva, S., Reyes-Lecca, R.C., Pinheiro, T.R. & Lacerda, M.V. Malaria is associated with poor school performance in an endemic area of the Brazilian Amazon. *Malar J*. **8**, 230 10.1186/1475-2875-8-230 (2009).

Tapajós, R., Castro, D., Melo, G., Balogun, S., James, M., Pessoa, R., Almeida, A., Costa, M., Pinto, R., Albuquerque, B., Monteiro, W., Braga, J., Lacerda, M. & Mourão, M. P. Malaria impact on cognitive function of children in a peri-urban community in the Brazilian Amazon. *Malar J*. **18**(1), 173 10.1186/s12936-019-2802-2 (2019).

Additionally, the Authors omitted the below references:

Brasil, L. M. B. F., Vieira, J. L. F., Araújo, E. C., Piani, P. P. F., Dias, R. M., Ventura, A. M. R. S., Cabral, B. C., Santa Brígida, R. C. R. & de Andrade, M. A. Cognitive performance of children living in endemic areas for *Plasmodium vivax*. *Malar. J*. **16**(1), 370 10.1186/s12936-017-2026-2 (2017).

Fernando, S. D., Gunawardena, D. M., Bandara, M. R., De Silva, D, Carter, R., Mendis, K. N. & Wickremasinghe, A. R. The impact of repeated malaria attacks on the school performance of children. *Am. J. Trop. Med. Hyg*. **69**(6), 582–588 (2003).

Halliday, K. E., Karanja, P., Turner, E. L., Okello, G., Njagi, K., Dubeck, M. M., Allen, E., Jukes, M. C. & Brooker, S. J. *Plasmodium falciparum*, anaemia and cognitive and educational performance among school children in an area of moderate malaria transmission: Baseline results of a cluster randomized trial on the coast of Kenya. *Trop. Med. Int. Health* **17**(5), 532–549 10.1111/j.1365-3156.2012.02971 (2012).

Carter, J. A., Mung'ala-Odera, V., Neville, B. G., Murira, G., Mturi, N., Musumba, C. & Newton, C. R. Persistent neurocognitive impairments associated with severe falciparum malaria in Kenyan children. *J. Neurol. Neurosurg. Psychiatry* **76**(4), 476–481 (2005).

Vorasan, N., Pan-Ngum, W., Jittamala, P., Maneeboonyang, W., Rukmanee, P. & Lawpoolsri, S. Long-term impact of childhood malaria infection on school performance among school children in a malaria endemic area along the Thai-Myanmar border. *Malar. J.* **14**(1), 401 10.1186/s12936-015-0917-7 (2015).

Ssenkusu, J.M., Hodges, J.S., Opoka, R.O., Idro, R., Shapiro, E., John, C.C. & Bangirana, P. Long-term behavioral problems in children with severe malaria. *Pediatrics* **138**(5), e20161965 10.1542/peds.2016-1965 (2016).

“Neuroinflammation after PBA infection of mice influences neurotrophin expression, which impairs hippocampal neurogenesis and increases hippocampal cell death. This is associated with impaired memory, but specifically short-term memory after the CM course^48^.”

Reference 48 was incorrectly given as:

Souza, J. B. D. E., Hafalla, J. C. R., Riley, E. M. & Couper, K. N. Cerebral malaria: Why experimental murine models are required to understand the pathogenesis of disease. *Parasitology* **137**, 755–772 (2010).

The correct reference is listed below:

de Miranda, A. S., Brant, F., Campos, A. C., Vieira, L. B., Rocha, N. P., Cisalpino, D., Binda, N. S., Rodrigues, D. H., Ransohoff, R. M., Machado, F. S., Rachid, M. A. & Teixeira, A. L. Evidence for the contribution of adult neurogenesis and hippocampal cell death in experimental cerebral malaria cognitive outcome. *Neuroscience* **284**, 920–933 10.1016/j.neuroscience.2014.10.062 (2015).

“[…] but they may be associated with cytokine storms during acute infection^51^, the production of neurotoxins by infected red blood cells^52^, impaired coagulation that can impact the correct oxygen supplementation to the tissues of the central nervous system^53^, anemia^54^, multiple infections^55^.”

Reference 52 was incorrectly given as:

Kihara, M., Carter, J. A. & Newton, C. R. J. C. The effect of *Plasmodium falciparum* on cognition: A systematic review. *Trop. Med. Int. Health 11*(4), 386–397. 10.1111/j.1365-3156.2006.01579.x (2006).

The correct reference is listed below:

Eugenin, E. A., Martiney, J. A. & Berman, J. W. The malaria toxin hemozoin induces apoptosis in human neurons and astrocytes: Potential role in the pathogenesis of cerebral malaria. *Brain Res*. **1720**, 146317 10.1016/j.brainres.2019.146317 (2019).

In addition, Reference 68 was removed.

68. Khalifa, A. Antiinfective agents affecting cognition: A review. *J. Chemother.* **19**(6), 620–631 (2007).

“ […] in the hippocampus of the mouse (81) and this can reduce cognitive impairment.”

Number 81 was removed.

The correct reference is listed below:

Cui, C. M., Gao, J. L., Cui, Y., Sun, L. Q., Wang, Y. C., Wang, K. J. *et al*. Chloroquine exerts neuroprotection following traumatic brain injury via suppression of inflammation and neuronal autophagic death. *Mol. Med. Rep*. [*Internet*]. **12**(2), 2323–2328 (2015). Accessed 21 Jul 2023.

Reference 52 and 68 were incorrectly given as:

Eugenin, E. A., Martiney, J. A. & Berman, J. W. The malaria toxin hemozoin induces apoptosis in human neurons and astrocytes: Potential role in the pathogenesis of cerebral malaria. *Brain Res*. **1720**, 146317 10.1016/j.brainres.2019.146317 (2019).

These references have been deleted.

As a result of the changes, the References have been renumbered.

Finally, this Article contained an error in Figure 1, where in the cases the patients exposed to malaria in T8 was 60, and in control the exclude between T2 and T8 was 5 and in T8 the patient not exposed to malaria in T8 was 64. The original Figure 1 and accompanying legend appear below.

**Figure 1:**
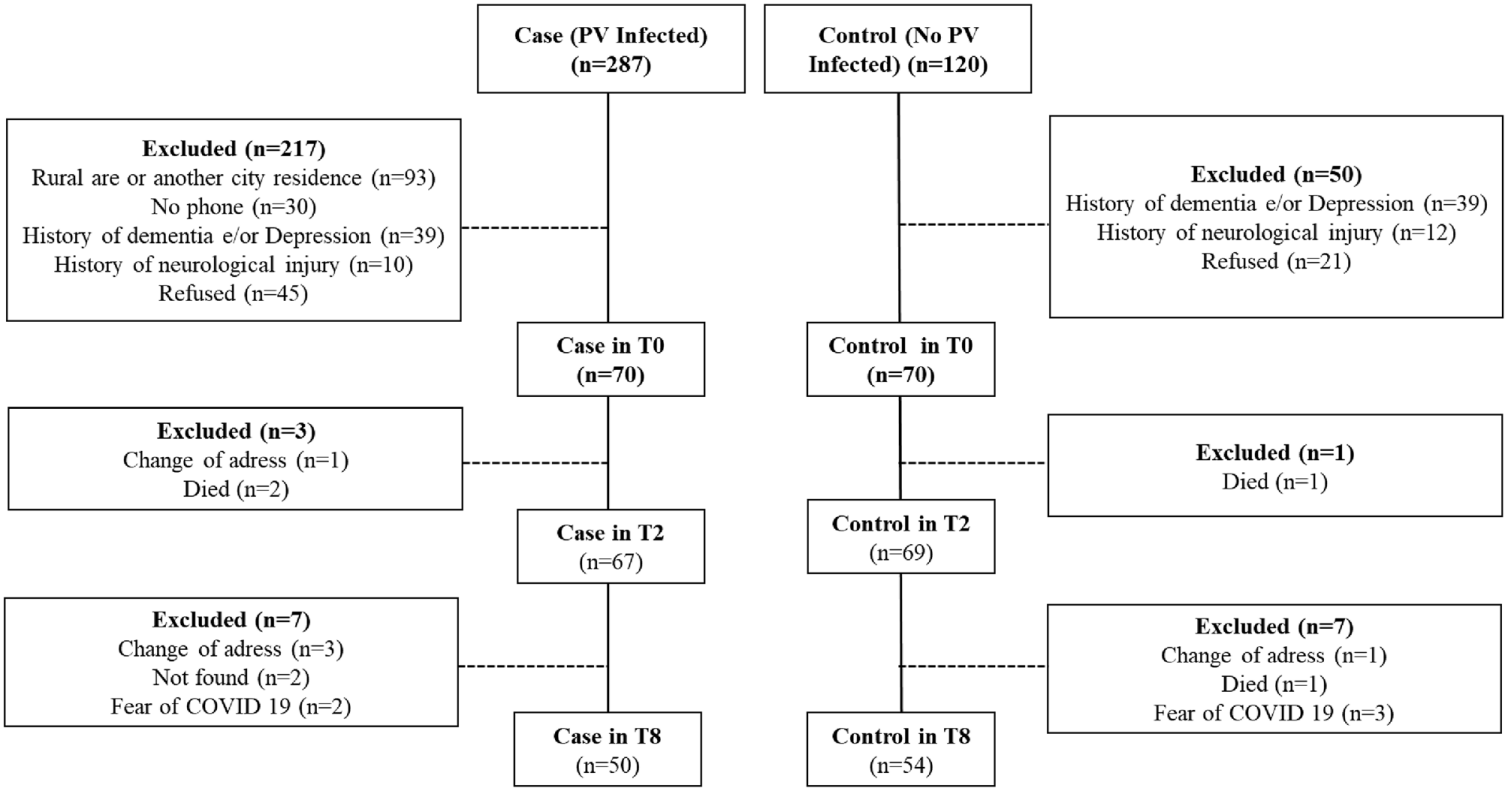
Flowchart of the study.

The original Article has been corrected.

