# Supplementary material for: Impact of Plasmodium vivax malaria on executive and cognitive functions in elderlies in the Brazilian Amazon
Source: Sci Rep. 2022 Jun 20;12:10361. doi: 10.1038/s41598-022-14175-0 (PMC9208538; doi:10.1038/s41598-022-14175-0)
Supplement: Supplementary file 1 — Supplementary Tables. [file 41598_2022_14175_MOESM1_ESM.docx]

**Supplementary Table 1:** Instruments and their respective domains evaluated

| **Instruments** | **Evaluated domains** |
| --- | --- |
| MMSE | Time orientation  Spatial orientation  Immediate and delayed memory  Attention  Calculation  Language  Constructive praxis  Praxis |
| CDT | Visuospatial skills  Constructive praxis  Executive functions  Attention |
| WAIS-III | Attention  Executive functions  Working memory  Episodic memory  Calculation  Language |
| BDI-II | Depression |
| WCST | Executive functions |

**Supplementary Table 2:** Previous infections, recurrences and parasitemia of exposed group

|  | |
| --- | --- |
| Characteristics | Exposed Group  n=70 |
| Previous infections by malaria, since 2010 until 2017, % | 30/70 (42.9%) |
| Recurrences until T2, % (1 or more) | 4/70 (5.7%) |
| Recurrence infections until T8, % (1 or more) | 17/70 (24.3%) |
| Parasitemia at T0, % (Higher than 500 parasites/mm3) | 47/68 (69.1%) |

**Supplementary Table 3:** Changes in cognitive and executive functions assessed by different tools in the week of malaria infection and post 2 and 8 months of infection. Uni and Multivariate Analysis

|  | Univariate Analysis | | | | Multivariate Analysis | | | |
| --- | --- | --- | --- | --- | --- | --- | --- | --- |
|  | Coef. | [95% Conf. Interval] | | *P* | Coef. | [95% Conf. Interval] | | *P* |
| **T0 (n = 140)** |  |  |  |  |  |  |  |  |
| Clock Drawing Test | **-0.21** | **-0.34** | **-0.08** | **0.00** | -0.14 | -0.28 | 0.00 | 0.06 |
| Mini-Mental State Examination | **-0.08** | **-0.14** | **-0.01** | **0.03** | -0.06 | -0.13 | 0.02 | 0.13 |
| **T2 (n = 136)** |  |  |  |  |  |  |  |  |
| Clock Drawing Test | **-0.31** | **-0.44** | **-0.18** | **0.00** | **-0.29** | **-0.43** | **-0.14** | **0.00** |
| Mini-Mental State Examination | **-0.13** | **-0.20** | **-0.06** | **0.00** | **-0.11** | **-0.19** | **-0.04** | **0.00** |
| WAIS | **-0.21** | **-0.28** | **-0.14** | **0.00** | **-0.18** | **-0.26** | **-0.10** | **0.00** |
| WCST - No. of categories completed | **-0.66** | **-0.91** | **-0.40** | **0.00** | **-0.66** | **-0.94** | **-0.37** | **0.00** |
| WCST - Total No. Correct | **-0.18** | **-0.22** | **-0.13** | **0.00** | **-0.18** | **-0.23** | **-0.13** | **0.00** |
| WCST - Perseverative responses | **0.23** | **0.19** | **0.27** | **0.00** | **0.19** | **0.14** | **0.24** | **0.00** |
| WCST - Perseverative errors | **0.22** | **0.17** | **0.27** | **0.00** | **0.18** | **0.13** | **0.23** | **0.00** |
| WCST – Nonperseverative errors | **0.00** | **-0.08** | **0.07** | **0.93** |  |  |  |  |
| WCST – Percent conceptual level responses | **-0.40** | **-0.47** | **-0.34** | **0.00** | **-0.42** | **-0.49** | **-0.35** | **0.00** |
| WCST - Failure to maintain set | -0.08 | -0.47 | 0.30 | 0.67 |  |  |  |  |
| **T8 (n=124)** |  |  |  |  |  |  |  |  |
| Clock Drawing Test | **-0.26** | **-0.39** | **-0.12** | **0.00** | **-0.19** | **-0.34** | **-0.04** | **0.01** |
| Mini-Mental State Examination | **-0.13** | **-0.20** | **-0.05** | **0.00** | **-0.12** | **-0.20** | **-0.04** | **0.00** |
| WAIS | **-0.29** | **-0.37** | **-0.21** | **0.00** | **-0.24** | **-0.33** | **-0.16** | **0.00** |
| WCST - No. of categories completed | **-0.66** | **-0.92** | **-0.40** | **0.00** | **-0.70** | **-0.99** | **-0.41** | **0.00** |
| WCST - Total No. Correct | **-0.25** | **-0.29** | **-0.20** | **0.00** | **-0.25** | **-0.30** | **-0.20** | **0.00** |
| WCST - Perseverative responses | **0.33** | **0.29** | **0.38** | **0.00** | **0.34** | **0.29** | **0.39** | **0.00** |
| WCST - Perseverative errors | **0.31** | **0.26** | **0.36** | **0.00** | **0.31** | **0.26** | **0.36** | **0.00** |
| WCST – Nonperseverative errors | -0.07 | -0.16 | 0.02 | 0.12 | -0.09 | -0.19 | 0.02 | 0.10 |
| WCST – Percent conceptual level responses | **-0.51** | **-0.57** | **-0.44** | **0.00** | **-0.51** | **-0.58** | **-0.44** | **0.00** |
| WCST - Failure to maintain set | **-0.51** | **-0.84** | **-0.18** | **0.00** | **-0.43** | **-0.80** | **-0.07** | **0.02** |

**Supplementary Table 4:** Comparison between parasitemia level groups (500 or less parasites/mm3, higher than 500 parasites/mm3) at T0 performance in cognitive and executive function. Uni and Multivariate Analysis

| **Parasitemia at T0**  **(500 or less parasites/mm3, Higher than 500 parasites/mm3)** | Univariate | | | | Multivariate | | | |
| --- | --- | --- | --- | --- | --- | --- | --- | --- |
|  | Coef. | [95% Conf. Interval] | | *P* | Coef. | [95% Conf. Interval] | | *P* |
| **T0 (n = 68)** |  |  |  |  |  |  |  |  |
| Clock Drawing Test | -0.07 | -0.28 | 0.14 | 0.52 |  |  |  |  |
| Mini-Mental State Examination | -0.03 | -0.14 | 0.07 | 0.53 |  |  |  |  |
| **T2 (n = 65)** |  |  |  |  |  |  |  |  |
| Clock Drawing Test | -0.09 | -0.31 | 0.12 | 0.40 |  |  |  |  |
| Mini-Mental State Examination | -0.06 | -0.16 | 0.05 | 0.31 |  |  |  |  |
| WAIS | -0.07 | -0.19 | 0.05 | 0.23 |  |  |  |  |
| WCST - No. of categories completed | -0.32 | -0.75 | 0.11 | 0.15 | -0.26 | -0.69 | 0.17 | 0.24 |
| WCST - Total No. Correct | **-0.15** | **-0.22** | **-0.08** | **0.00** | **-0.13** | **-0.21** | **-0.06** | **0.00** |
| WCST - Perseverative responses | 0.04 | -0.02 | 0.11 | 0.16 | 0.03 | -0.03 | 0.10 | 0.28 |
| WCST - Perseverative errors | 0.03 | -0.04 | 0.10 | 0.44 |  |  |  |  |
| WCST – Nonperseverative errors | **0.29** | **0.16** | **0.41** | **0.00** | **0.27** | **0.15** | **0.40** | **0.00** |
| WCST – Percent conceptual level responses | **-0.22** | **-0.33** | **-0.11** | **0.00** | **-0.18** | **-0.29** | **-0.08** | **0.00** |
| WCST - Failure to maintain set | -0.57 | -1.13 | -0.01 | 0.05 | -0.55 | -1.11 | 0.01 | 0.05 |
| **T8 (n=59)** |  |  |  |  |  |  |  |  |
| Clock Drawing Test | 0.05 | -0.18 | 0.27 | 0.68 |  |  |  |  |
| Mini-Mental State Examination | -0.05 | -0.16 | 0.06 | 0.36 |  |  |  |  |
| WAIS | -0.08 | -0.20 | 0.04 | 0.21 |  |  |  |  |
| WCST - No. of trials administered | 0.02 | -0.03 | 0.06 | 0.52 |  |  |  |  |
| WCST - Total No. Correct | 0.01 | -0.07 | 0.09 | 0.79 |  |  |  |  |
| WCST - Perseverative responses | 0.00 | -0.06 | 0.06 | 0.91 |  |  |  |  |
| WCST - Perseverative errors | 0.00 | -0.06 | 0.07 | 0.89 |  |  |  |  |
| WCST – Nonperseverative errors | 0.08 | -0.06 | 0.22 | 0.26 |  |  |  |  |
| WCST – Percent conceptual level responses | -0.02 | -0.13 | 0.09 | 0.71 |  |  |  |  |
| WCST - Failure to maintain set | 0.31 | -0.28 | 0.91 | 0.30 |  |  |  |  |

**Supplementary Table 5:** Comparison between previous infections groups (No infections, had infections) performance in cognitive and executive function. Uni and Multivariate Analysis

| **Previous infections**  **(No infections, had infections)** | Univariate | | | | Multivariate | | | |
| --- | --- | --- | --- | --- | --- | --- | --- | --- |
|  | Coef. | [95% Conf. Interval] | | *P* | Coef. | [95% Conf. Interval] | | *P* |
| **T0 (n = 70)** |  |  |  |  |  |  |  |  |
| Clock Drawing Test | 0.02 | -0.17 | 0.22 | 0.82 |  |  |  |  |
| Mini-Mental State Examination | 0.00 | -0.10 | 0.10 | 0.98 |  |  |  |  |
| **T2 (n = 67)** |  |  |  |  |  |  |  |  |
| Clock Drawing Test | **0.26** | **0.06** | **0.46** | **0.01** | 0.17 | -0.04 | 0.39 | 0.12 |
| Mini-Mental State Examination | 0.03 | -0.07 | 0.13 | 0.58 |  |  |  |  |
| WAIS | 0.09 | -0.02 | 0.20 | 0.10 | 0.03 | -0.09 | 0.14 | 0.67 |
| WCST - No. of categories completed | -0.31 | -0.74 | 0.13 | 0.17 | **-0.50** | **-0.96** | **-0.03** | **0.04** |
| WCST - Total No. Correct | -0.04 | -0.11 | 0.02 | 0.20 | -0.07 | -0.15 | 0.00 | 0.05 |
| WCST - Perseverative responses | **0.06** | **0.01** | **0.12** | **0.03** | **0.07** | **0.01** | **0.13** | **0.02** |
| WCST - Perseverative errors | 0.05 | -0.01 | 0.11 | 0.12 | 0.06 | -0.01 | 0.13 | 0.09 |
| WCST – Nonperseverative errors | 0.03 | -0.08 | 0.14 | 0.61 |  |  |  |  |
| WCST – Percent conceptual level responses | **-0.14** | **-0.25** | **-0.04** | **0.01** | **-0.22** | **-0.33** | **-0.11** | **0.00** |
| WCST - Failure to maintain set | 0.11 | -0.45 | 0.67 | 0.70 |  |  |  |  |
| **T8 (n=60)** |  |  |  |  |  |  |  |  |
| Clock Drawing Test | **0.25** | **0.04** | **0.46** | **0.02** | 0.13 | -0.09 | 0.35 | 0.26 |
| Mini-Mental State Examination | 0.03 | -0.08 | 0.13 | 0.64 |  |  |  |  |
| WAIS | 0.05 | -0.07 | 0.17 | 0.40 |  |  |  |  |
| WCST - No. of categories completed | -0.07 | -0.50 | 0.37 | 0.76 |  |  |  |  |
| WCST - Total No. Correct | 0.03 | -0.04 | 0.10 | 0.37 |  |  |  |  |
| WCST - Perseverative responses | **-0.08** | **-0.14** | **-0.02** | **0.01** | -0.03 | -0.09 | 0.03 | 0.30 |
| WCST - Perseverative errors | **-0.07** | **-0.13** | **0.00** | **0.05** | -0.02 | -0.09 | 0.05 | 0.54 |
| WCST – Nonperseverative errors | **0.26** | **0.12** | **0.39** | **0.00** | **0.27** | **0.12** | **0.41** | **0.00** |
| WCST – Percent conceptual level responses | 0.04 | -0.07 | 0.14 | 0.51 |  |  |  |  |
| WCST - Failure to maintain set | 0.01 | -0.52 | 0.55 | 0.96 |  |  |  |  |

**Supplementary Table 6:** Comparison between recurrences groups (No recurrences, had recurrences) performance in cognitive and executive function. Uni and Multivariate Analysis

| **Recurrences until t8**  **(No recurrences, had recurrences)** | Univariada | | | | Multivariada | | | |
| --- | --- | --- | --- | --- | --- | --- | --- | --- |
|  | Coef. | [95% Conf. Interval] | | *P* | Coef. | [95% Conf. Interval] | | *P* |
| **T8 (n=6)** |  |  |  |  |  |  |  |  |
| Clock Drawing Test | -0.13 | -0.37 | 0.11 | 0.30 |  |  |  |  |
| Mini-Mental State Examination | -0.06 | -0.18 | 0.06 | 0.34 |  |  |  |  |
| WAIS | -0.08 | -0.21 | 0.06 | 0.26 |  |  |  |  |
| WCST - No. of categories completed | -0.02 | -0.51 | 0.46 | 0.92 |  |  |  |  |
| WCST - Total No. Correct | 0.01 | -0.07 | 0.09 | 0.79 |  |  |  |  |
| WCST - Perseverative responses | -0.05 | -0.11 | 0.02 | 0.17 | **-0.09** | **-0.16** | **-0.02** | **0.01** |
| WCST - Perseverative errors | -0.04 | -0.12 | 0.03 | 0.24 |  |  |  |  |
| WCST – Nonperseverative errors | 0.21 | 0.07 | 0.36 | 0.00 | **0.23** | **0.07** | **0.38** | **0.00** |
| WCST – Percent conceptual level responses | 0.05 | -0.07 | 0.17 | 0.42 |  |  |  |  |
| WCST - Failure to maintain set | 0.29 | -0.27 | 0.85 | 0.31 |  |  |  |  |
